# Action of Antimicrobial Peptides against Bacterial Biofilms

**DOI:** 10.3390/ma11122468

**Published:** 2018-12-05

**Authors:** Muhammad Yasir, Mark Duncan Perry Willcox, Debarun Dutta

**Affiliations:** School of Optometry and Vision Science, University of New South Wales, Sydney, NSW 2052, Australia; m.yasir@unsw.edu.au (M.Y.); debarun.dutta@unsw.edu.au (D.D.)

**Keywords:** biofilm, antimicrobial peptides, mechanism of action, medical devices, biomaterials

## Abstract

Microbes are known to colonize surfaces and form biofilms. These biofilms are communities of microbes encased in a self-produced matrix that often contains polysaccharides, DNA and proteins. Antimicrobial peptides (AMPs) have been used to control the formation and to eradicate mature biofilms. Naturally occurring or synthetic antimicrobial peptides have been shown to prevent microbial colonization of surfaces, to kill bacteria in biofilms and to disrupt the biofilm structure. This review systemically analyzed published data since 1970 to summarize the possible anti-biofilm mechanisms of AMPs. One hundred and sixty-two published reports were initially selected for this review following searches using the criteria ‘antimicrobial peptide’ OR ‘peptide’ AND ‘mechanism of action’ AND ‘biofilm’ OR ‘antibiofilm’ in the databases PubMed; Scopus; Web of Science; MEDLINE; and Cochrane Library. Studies that investigated anti-biofilm activities without describing the possible mechanisms were removed from the analysis. A total of 17 original reports were included which have articulated the mechanism of antimicrobial action of AMPs against biofilms. The major anti-biofilm mechanisms of antimicrobial peptides are: (1) disruption or degradation of the membrane potential of biofilm embedded cells; (2) interruption of bacterial cell signaling systems; (3) degradation of the polysaccharide and biofilm matrix; (4) inhibition of the alarmone system to avoid the bacterial stringent response; (5) downregulation of genes responsible for biofilm formation and transportation of binding proteins.

## 1. Biofilms

A biofilm is a group of organisms such as fungi, bacteria, and viruses, existing in a sessile form and surrounded by a self-produced extracellular matrix. Costerton et al. [1] proposed a basic definition of biofilm as “a structured community of bacterial cells enclosed in a self-produced polymeric matrix and adherent to an inert or living surface” and Hall-Stoodley et al. [2] defined biofilms as “surface-associated microbial communities, surrounded by an extracellular polymeric substance (EPS) matrix”. A biofilm can also be called “an aggregate of microbial cells adherent to a living or nonliving surface, embedded within a matrix of EPS of microbial origin” [3]. Recently, biofilms have been described as complex sessile communities of microbes found either attached to a surface or buried firmly in an extracellular matrix as aggregates [4]. The matrix can be composed of exopolysaccharides, proteins, nucleic acids, and other cellular debris collectively called extra polymeric substances (EPS) [5,6,7]. 

### 1.1. Biofilm Formation

The process of biofilm formation on biomaterials begins by the initial adhesion of planktonic bacteria to surfaces and then aggregation into smaller groups of bacteria known as microcolonies. Following attachment, EPS such as proteins, glycoproteins, glycolipids, and extracellular DNA are synthesized [8]. Glycopeptides, glycolipids and lipopolysaccharides help to keep the biofilms intact [9]. In mature biofilms, the microcolonies differentiate into distinct phenotypes which are significantly different in gene expression than their planktonic counterparts [10]. The differentiation can be triggered by the accumulation of quorum sensing molecules such as N-acyl homoserine lactones that facilitate cell to cell communication [1].

Starvation conditions are known to promote the formation of biofilms, and bacteria grown or living under starvation are known to have higher antibiotic tolerance. Biofilm formation can be an adaptation of microorganisms to hostile environments [11,12]. Under hostile conditions bacteria can activate the stringent response (which can be characterized by the production of “alarmones”) by synthesizing the signaling nucleoside guanosine pentaphosphate or tetraphosphate ((p)ppGpp) which can cause the inhibition of RNA synthesis when amino acids are in low concentrations [13]. RelA and SpoT are homologous proteins that are responsible for modulating intracellular concentrations of (p)ppGpp, often conserved among Gram-negative and Gram-positive bacteria, with a few exceptions such as *S. aureus* [14]. This stringent response plays an important role in the development of biofilms as mutants lacking RelA and SpoT produce comparatively fragile and antibiotic sensitive biofilms [15]. The exact role of (p)ppGpp in biofilm formation is not known, but it is likely that hostile conditions trigger transcription of hundreds of genes responsible for altered intracellular metabolism and energy conservation through suspension of cell division [15]. 

Biofilm formation can occur on a variety of surfaces, including living tissues, medical devices, industrial or potable water system piping, or on surfaces in the natural aquatic environment [16]. Approximately 99% of the microbial world exists as biofilms [17,18] and these biofilms are diverse containing a wide range of microbes [19]. For example, more than 500 types of bacteria are present in biofilms in the oral cavity [20]. 

### 1.2. Characteristics of Biofilms

Biofilm embedded cells are not as sensitive to antimicrobials compared with their planktonic counterparts. [21] They are highly resistant to conventional antibiotics, up to 1000 times more than planktonic bacteria. This is related in part to the slow growth rate and low metabolic activity of cells in biofilms [15,22,23]. In addition, the EPS matrix surrounding biofilms, which can make up to 50% to 90% of the total biomass of biofilms, resists the penetration of antimicrobials [16,24,25,26,27,28,29,30]. Moreover, microbes in biofilms can have a high rate of mutation and exchange of resistance genes on mobile genetic elements [31,32] which can also lead to increase in the overall resistance of cells in biofilms.

### 1.3. Biofilm-Associated Infections

Biofilms pose a serious threat to public health because of their potential to cause biomaterial- associated infections due in-part to the high resistance of biofilms to antimicrobials agents [33]. About 80% of bacterial infections in humans are caused by biofilms [1,12,23]. Biofilm mediated infection can be divided into two categories, non-device and device associated infections [34,35]. 

The first category involves biofilm formation on host tissues such as epithelial, mucosal surfaces, and teeth. These can cause infections associated with cystic fibrosis (CF) patients, foot ulcers in diabetic patients, chronic otitis media or rhinosinusitis, chronic prostatitis, recurrent urinary tract infections, and dental caries and periodontitis [36,37]. 

The second category of infections arises due to the microbial colonization of abiotic objects, for example indwelling medical devices such as central venous or urinary catheters, joint or dental prostheses, heart valves, endotracheal tubes, intrauterine devices, and dental implants [34,35,38]. Microbes can detach from these biofilms and disseminate to the surrounding tissues or to the bloodstream, further exacerbating the infection [39]. Worldwide production of biomedical devices and tissue engineering-related objects is approximately $180 billion per annum [37]. According to current estimates, over 5 million medical devices or implants are used annually in the U.S.A. alone [37]. About 60–70% of nosocomial infections are associated with biomaterials or implants [37]. Regardless of the sophistication of the biomedical implant and tissue engineering constructs, all medical devices are susceptible to microbial colonization and can cause infections [40,41,42]. Biofilm growth on medical devices can be extremely difficult to eradicate, with only a few treatment options such as removal of the infected device or use of large doses of antibiotics [43]. However, this increases treatment costs and may increase the potential for the development of antibiotic resistance and cytotoxicity [44]. Moreover, removal may not be an easy option for patients with medical devices for critical care such as pacemakers. The clinical significance of biofilm-associated infections and their inherent resistance to antimicrobials urgently demand development of novel anti-biofilm compounds. 

## 2. Antimicrobial Peptides

Antimicrobial peptides (AMPs) have a varying number (from five to over a hundred) of amino acids, most commonly L forms, with molecular weights between 1–5 KDa. AMPs have a broad spectrum of activity ranging from viruses to parasites [45]. AMPs are generally cationic in nature, and often referred as cationic host defense peptides because of their role in the immune response [46]. They are also known as cationic amphipathic peptides [47], cationic AMPs [48], and α-helical AMPs [49]. Recently, a few anionic antimicrobial peptides have been reported which have a net charge ranging from −1 to −7, and a length from 5 to circa 7 amino acid residues [50]. AMPs have been recognized as promising alternatives to conventional antibiotics due to their multiple target sites and non-specific mechanism of action which reduces the chances of resistance development. AMPs exhibit strong anti-biofilm activity against multidrug resistant as well as clinically isolated bacterial biofilms [51]. AMPs can interfere in the early stages of biofilm formation to prevent the initial adhesion of bacteria to surfaces [51]. They can destroy mature biofilms by encouraging microbial detachment or killing [52]. Here we focus on the anti-biofilm action of AMPs against different Gram-positive and Gram-negative bacteria, with emphasis on their mechanism of action. 

Based on their secondary structure, AMPs are generally categorized into four groups (1) α-helical AMPs; (2) β-sheet AMPs; (3) extended AMPs; and (4) cationic loop AMPs [53]. Alpha-helical peptides are the largest group of AMPs representing 30–50% of all AMPs of known secondary structure [54,55,56]. These peptides commonly consist of 12–40 amino acids and contain an abundance of helix stabilizing amino acids such as alanine, leucine, and lysine [56]. Beta-sheet AMPs usually consist of two to ten cysteine residues that from one to five inter-chain disulfide bonds that help the peptides to form the beta-sheet [57]. Beta-sheet antimicrobial peptides include the defensin family of peptides [58,59]. Defensins consist of two to three antiparallel beta-sheets however, in some cases alpha-helical or unstructured segments can be found at their N- or C-termini [60]. Compared with α-helical antimicrobial peptides, the defensins adopt a globular structure in aqueous solutions [60,61]. Despite extensive variations in length, amino acid composition and net positive charge, β-strands are observed in all α- and β-defensins [62,63]. Extended AMPs are not folded into α-helix or β-sheet structures. These AMPs often contain high numbers of arginine, tryptophan, proline or cystine residues [64]. Some of these AMPs can fold into defined amphipathic molecules in bacterial membranes, but often these are not membrane active [65]. The proline-rich insect-derived pyrrhocoricin, drosocin and apidaecin peptides penetrate membranes and exert their antimicrobial activities by interacting with intracellular proteins such as the heat-shock protein DnaK and GroEL to inhibit the DnaK ATPase and chaperone-assisted protein folding related activities, respectively [66,67]. Cationic loop AMPs are proline-arginine rich peptides, and because of their high numbers of proline residues, they rarely form amphipathic characteristics and tend to adopt polyproline helical type-II structures [68].

### 2.1. Mechanism of Action of AMPs against Planktonic Bacteria

The mechanism of action of AMPs usually starts by interacting with negatively charged moieties such as lipopolysaccharides (LPS) in the outer membranes of Gram-negative bacteria and lipoteichoic acid (LTA) in the cell wall of Gram-positive bacteria [69,70,71]. Once AMPs cross or produce pores in the outer membrane or the cell wall of bacteria, disruption of cytoplasmic membranes occurs followed by cell lysis [72]. The mechanisms of action of AMPs have been divided into pore-forming and non-pore models [73]. Pore-forming models include the barrel stave and the toroidal pore models. Non-pore models include the carpet model. AMPs can also inhibit the synthesis of cell walls, DNA, RNA and protein, and activate enzymes such as autolysins that induce autolytic death [66,74,75].

### 2.2. Mechanism of Action against Biofilms

In this review, we systemically analyzed all published data since 1970 to summarize all the possible anti-biofilm mechanisms of antimicrobial peptides. A total of 162 published reports were initially selected for this review following search criteria using ‘antimicrobial peptide’ OR ‘peptide’ AND ‘mechanism of action’ AND ‘biofilm’ OR ‘antibiofilm’ in the databases PubMed, Scopus, Web of Science, MEDLINE, and Cochrane Library. The studies investigated the antimicrobial activity of AMPs against a variety of microorganisms. A total of 17 original reports qualified for our review which have articulated the mechanism of anti-biofilm action of AMPs. These reports are included in this review.

Several overlapping anti-biofilm mechanisms of AMPs are reported in the literature. Following careful consideration, we found five major anti-biofilm mechanisms: (1) disruption or degradation of the membrane potential of biofilm embedded cells; (2) interruption of bacterial cell signaling systems; (3) degradation of the polysaccharide and biofilm matrix; (4) inhibition of the alarmone system to avoid the bacterial stringent response; (5) downregulation of genes responsible for biofilm formation and transportation of binding proteins. 

**Figure 1:**
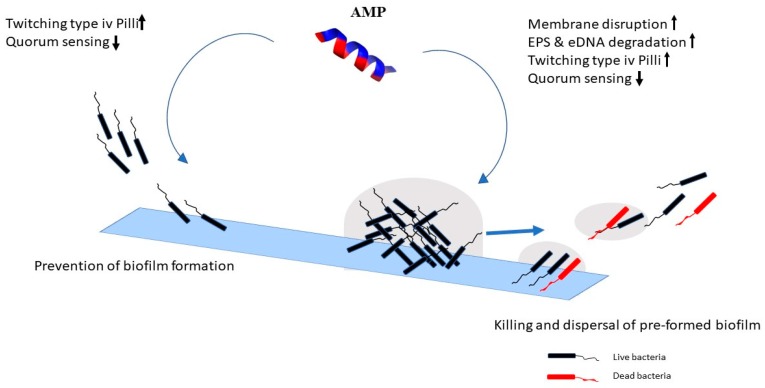
Anti-biofilm activity and mechanism of action of antimicrobial peptides (AMPs). AMPs effect mainly involve prevention of bacterial attachment and inhibition of biofilm formation or disruption of pre-formed biofilms. 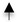 activation 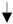 inhibition.

Certain synthetic AMPs can rapidly degrade pre-established biofilms of *P. aeruginosa* [52]. Although the mechanism of biofilm degradation is poorly understood, the rapid destruction of biofilm embedded cells [52] may indicate that they act by disrupting the membranes of the bacteria. Table 1 and Figure 1 summarize the mechanisms of biofilm inhibition and degradation of various AMPs. Mechanistic studies have tended to focus on the membrane-disrupting properties of AMPs [76,77]. 

(i) disruption or degradation of the membrane potential of biofilm embedded cells

Three bacteriocins (nisin A, lacticin Q, and nukacin ISK-1) can destroy the membrane potential of biofilm embedded cells of *S. aureus* (an MRSA strain) and can cause the release of ATP from the cells [78]. An engineered peptide RN3(5-17P22-36) [79] derived from the cationic proteins of eosinophil granules [80,81] can kill bacteria via membrane disruption. However, this membrane depolarization of cells in biofilms was 2–3-fold less compared with planktonic bacteria at the same concentration [79]. A frog skin-derived AMP esculentin (Esc (1-21) can permeabilize the cytoplasmic membrane of *P. aeruginosa* PAO1 in biofilms and cause release of β-galactosidase [82]. However, this effect was slower and did not result in comparable β-galactosidase release compared to its action on planktonic cells [82]. The AMP (CSA)-13 can quickly penetrate into biofilms and permeabilize the cell membranes of biofilm cells of *P. aeruginosa* [83]. 

(ii) interruption of the bacterial cell signaling system

Human cathelicidic LL-37 and indolicidin can prevent biofilm formation of *P. aeruginosa* possibly by down-regulating the transcription of two major quorum-sensing systems, Las and Rhl [84]. Another mechanism by which AMPs have been shown to inhibit the formation of biofilms is by increasing twitching motility in *P. aeruginosa* by stimulating the expression of genes needed for type IV pilli biosynthesis and function [84,85]. The main function of type IV pilli is to increase the movement of bacteria on surfaces, which may facilitate removal of cells [86]. 

(iii) degradation of the polysaccharide and biofilm matrix

AMPs can also act on the extracellular polymeric matrix of bacterial biofilms. For example, peptide PI can degrade the EPS produced by *Streptococcus mutans* leading to reductions in biofilms formed on polystyrene or and saliva-coated hydroxyapatite [87]. An anti-biofilm peptide derived from maggots of the blowfly *Calliphora vicina* can degrade the biofilm matrix produced by drug resistant *Escherichia coli*, *Staphylococcus aureus* and *Acinetobacter baumannii* but the mechanism of degradation was not investigated [88]. Human liver-derived antimicrobial peptide hepcidin 20 can reduce the mass of extracellular matrix and alter the architecture of biofilms of *S. epidermidis* by targeting polysaccharide intercellular adhesin (PIA) [89]. Another peptide S4(1–16) M4Ka, a derivative of S4, has been shown to act against immature *P. aeruginosa* biofilms by disintegration and release of membrane lipids, detachment of bacteria and inhibition of biofilm formation [90]. The fish derived AMP piscidin-3 has nucleosidase activity and can destroy extracellular DNA of *P. aeruginosa* by coordinating with Cu^2+^ through its N-terminus [91]. 

(iv) inhibition of the alarmone system to avoid the bacterial stringent response

Anti-biofilm peptides may act by targeting an almost universal stringent stress response in both Gram-positive and Gram-negative bacteria [92]. Many bacteria produce the signaling nucleotides guanosine 5′-diphosphate 3′-diphosphate (ppGpp) and (p)ppGpp, that can regulate the expression of a plethora of genes [93,94] and are important in biofilm formation [95]. The AMPs 1018, DJK-5, and DJK-6 can block the synthesis and trigger degradation of (p)ppGpp in both Gram-positive and Gram-negative bacteria, and this can lead to reduction in biofilm formation which in turn increases susceptibility to AMPs [15]. Some other AMPs such as DJK-5 and 1018 can act on the stringent response in *P. aeruginosa* by suppressing spoT promoter activity [96]. DJK-5 and DJK-6 can degrade (p)ppGpp on *P. aeruginosa* biofilms to higher extent than 1018 [14].

(v) downregulation of genes responsible for biofilm formation and transportation of binding proteins 

**Figure 2:**
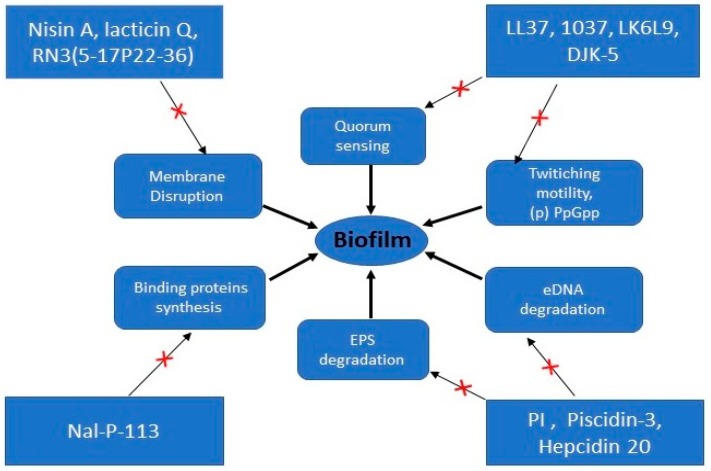
Representation of the different targets of anti-biofilm AMPs. × signs indicate inhibition and/or action on targets sites.

Biofilm formation by staphylococci is an accumulative process which crucially depends upon the synthesis of polysaccharide intercellular adhesin molecule PIA encoded by icaADBC locus in staphylococci [97]. Human β-defensin 3 (hBD-3) can reduce the expression of icaA, icaD and icaR genes of *Staphylococus epidermidis* ATCC 35,984 thereby reducing biofilm formation [98]. AMPs can inhibit genes controlling the mobility of extrachromosomal elements and transport and binding proteins [99]. A peptide Nal-P-113, can inhibit *Porphyromonas *gingivalis** biofilm formation by down-regulating genes such as PG0282 and PG1663 which encode ABC transporter and ATP-binding protein [99]. ABC transporters have been involved in cell-to-surface and cell-to-cell interactions in biofilms formation [100,101]. Figure 2 summarizes the targets sites of representative anti-biofilm AMPs.

## 3. Biofilm Resistance to AMPs

### 3.1. Interaction with EPS

It is thought that biofilm mediated resistance to AMPs is mainly due to their interaction with EPS, however the exact mechanism of interaction remained unknown in large number of cases [102]. Although most of the substances in EPS are negatively charged, the positively charged exopolymer PIA (which is composed of poly-*N*-acetyl glucosamine) can cause electrostatic repulsion of the cationic AMPs [103]. PIA protects *S. epidermidis* and *S. aureus* from the bactericidal actions of cationic AMPs such as LL-37 and human β-defensin [103]. PIA can also protect bacteria in biofilm from anionic AMP such as dermcidin (a human epithelial secreted) [102]. So, the role of PIA in protection of bacterial biofilms may be due to sequestration of AMPs along with electrostatic repulsion [102].

Gram negative bacteria such as *P. aeruginosa* secrete an anionic extracellular polysaccharide known as alginate which is made up of the uronic acid D-mannuronate and C-5 epimer-L guluronate [104,105]. Alginate can interact with positively charge AMPs and protect *P. aeruginosa* biofilm embedded cells from attack of AMPs [106]. Wild-type strains such as PAO1, PA14 (a mucoid cystic fibrosis strain), and FRD1 (a mutant which lacks alginate producing ability) can be easily killed by human leukocytes and their peptides within 4 h of exposure, [107] but became resistant in the presence of alginate [107]. Alginate can bind and induce an α-helical conformation for AMPs such as magainin II and cecropin P1 which is similar to their interaction with cytoplasmic membranes, suggesting that alginate can mediate hydrophobic interactions with AMPs despite its hydrophilic nature [106]. Alginate can trap AMPs in hydrophobic microdomains which consist of pyranosyl C–H groups that are inducible upon formation of AMPs-alginate complexes due to charge neutralization between the two species [108]. However, with the exception of cystic fibrosis, mucoid strains of *P. aeruginosa* account for only 1% of isolates from infections [109] so the role of mucoid strains in medical device related infections is limited. In contrast to mucoid strains, non-mucoid strains contain low levels of alginate [110] but can use either Pel or Psl (a structural cationic exopolysaccharide) to develop biofilms [111].

### 3.2. Adaptive Resistance Mechanism

Staphylococci have a peptide sensing system known as *aps*, which was first recognized in *S. epidermidis* [112]. The *aps* consist of two-component system that has a sensor histidine kinase (ApsS) and a DNA-binding response regulator (ApsR). A third component (ApsX) is also found only in some staphylococci species [112]. This *aps* system can protect Gram positive bacteria including methicillin resistant *S. aureus* (MRSA) strains from action of AMPs [113]. The aps system upregulates D-alanylation of teichoic acid and increases the expression of putative AMP efflux pumps [114]. A D-alanine deficient mutant of *E*. *faecalis* produced less biofilm but was more resistant to AMPs than the wild type [115]. The PhoP/PhoQ genetic system found in *P. aeruginosa* and *Salmonella enterica* [116] is used to sense AMPs [117]. This system tends to change the structure of LPS by addition of aminoarabinose to lipid A, which has the effect of decreasing the net negative charge of lipopolysaccharides [118]. Therefore, this system may also confer resistance of biofilm bacteria to AMPs. A two-component regulatory system pmrA-pmrB identified in *P. aeruginosa* that regulates resistance to polymyxin B, polymyxins E, cattle indolicidin and LL-37 [119] modifies lipopolysaccharides in the outer membrane of bacteria and this reduces the AMPs interaction with the outer membrane [120,121] this confering resistance.

### 3.3. Heterogeneity

Biofilms consist of structurally and functionally diverse bacterial populations and maintain a micro-environment which controls microbial activity, intracellular signaling and metabolic and genetic material exchange [122]. These properties can establish cellular and communal behaviors which result in tolerance and persistence of cells in the presence of antimicrobials [122]. For example, colistin can kill low metabolically active *P. aeruginosa* in biofilms but cannot destroy metabolically active cells [123]. This resistance to colistin in biofilms may be due to physiological tolerance [124]. *E. coli* possessing IncF plasmids can differentiate into structured and unstructured biofilms and can produce genetically regulated tolerant subpopulations [124]. Colistin can kill a small number of genetically tolerant bacteria in structured biofilms but can kill a high number of bacteria in unstructured biofilms. [124]. 

### 3.4. Synergy of Anti-Biofilm AMPs with Antibiotics

The anti-biofilm activity of AMPs can be enhanced against biofilms by combining them with antibiotics [125,126,127,128]. Combination strategies are useful since they can target a variety of microbial communities present with different metabolisms cells in low pH, hypoxic or low nutritious environments [129]. AMP-1018 can prevent initial bacterial attachment to surfaces by inhibiting the synthesis of (p)ppGpp [23]. When 1018 was used in combination with ceftazidime, ciprofloxacin, imipenem, or tobramycin, at sub-MIC this combination could inhibit 50% biofilms produced by *P. aeruginosa*, *E. coli*, *A. *baumannii**, *K. pneumoniae, S. *enterica**, and methicillin-resistant *S. aureus (MRSA)* [23]. Similarly, colistin in combination with temporin A (TEMP-A), citropin 1.1 (CIT-1.1) and tachyplesin I (TP-I-L) can eradiate mature biofilms of drug resistant *P. aeruginosa* and *S. aureus* [130]. AMPs can act synergistically with antibiotics against biofilm following two types of mechanism. Firstly, AMPs-antibiotic combinations can degrade biofilms matrix then AMPs act alone and disperse biofilms embedded cells [131]. AMP-antibiotic combinations can also be used against fungal biofilms [132]. An antifungal plant defensin derived peptide HsLin06_18 acts synergistically with caspofungin against *Candida glabrata* and *Candida albicans*. HsLin06_18 was shown to act by permeabilization cell membrane which facilitated caspofungin penetration into the fungal cells, inducing death at a sub-inhibitory concentration [132].

## 4. Future Considerations

Treating bacterial infections caused by biofilm-producing microorganisms is a troublesome task and a major challenge for health care systems. Antibiotic therapy or antibiotic releasing products are not adequate to control biofilm related infections, particularly due to the emergence of antibiotic resistant infections. Currently, there is no clear answer for the management and prevention of these infections. Use of very high concentrations of antibiotics in attempts to disrupt or prevent biofilm formation can be associated with cytotoxicity and poor prognosis. Hence, finding an alternative class of drugs to address biofilm-related infections represents a promising strategy. AMPs have broad-spectrum antimicrobial activity and are generally immune to development of bacterial resistance [45,133] and can work synergistically with first line antibiotics. AMPs have several promising characteristics that can be used to inhibit biofilms. However, there is limited information on the interaction of AMPs with biofilm components. More research is needed to understand their precise mechanisms of action such as inhibiting QS signals that restrict biofilm formation and interfere with signaling pathways involved in the synthesis of EPS. Molecular modelling approaches may provide insights on action of AMPs on biofilms. AMP-AMP and AMP-drug combinations that can induce biofilm matrix degradation could be the potential areas of future anti-biofilm research. 

In conclusion, this review found that AMPs have a variety of active anti-biofilm mechanisms that could be exploited for clinical applications to eradicate biofilms. It is clear that AMPs have high potential for further development as an active anti-biofilm agent, particularly in the high-risk environments such as hospital settings. AMPs could be used as a stand-alone therapy or in combination with other antimicrobials to eradicate biofilms. Further in vivo investigations are warranted to better understand the complex host environment that may affect their efficacy by reducing their activity and stability. Moreover, the role of immunomodulatory activities must be evaluated in complex biofilm environment in vivo. 

## Figures and Tables

**Table 1:** Representative AMPs and their anti-biofilm mechanism of action

AMPs	Sources	Amino Acids Sequence	Microorganisms	Proposed Mechanism of Action	Ref
LL-37	Human	LLGDFFRKSKEKIGKEFKRIVQRIKDFLRNLVPRTES	*Pseudomonas aeruginosa*	Reduces swimming and swarming motilities, promotes twitching motility, downregulates the genes required for biofilm formation and influences QS system	[84,85]
1037	Denovo	KRFRIRVRV	*Pseudomonas aeruginosa*		
1018	Denovo	VRLIVAVRIWRR	*Pseudomonas aeruginosa*	Decrease intracellular (p) PpGpp	[14]
Esculentin-1a (1–21)	Denovo	GIFSKLAGKKIKNLLISGLKG	*Pseudomonas aeruginosa*	Disrupts cell membrane	[82]
Nisin A	Denovo	MSTKDFNLDLVSVSKKDSGASPR	*Staphylococcus aureus*	Depolarizes cell membrane	[78]
lacticin Q	Denovo	MAGFLKVVQLLAKYGSKAVQMAWANKGKILDWLNAGQAIDKVVSKIKQILGIK	*Staphylococcus aureus*	Depolarizes cell membrane	[78]
Nukacin ISK-1	Denovo	KK-KSGVIPTVSHGCHMNSFQFVFTCC	*Staphylococcus aureus*	Depolarizes cell membrane	[78]
RN3(5-17P22-36)	Denovo	RPFTRAQWFAIQHISPRTIAMRAINNYRWR	*Pseudomonas aeruginosa*	Depolarizes and permeabilize cell membrane	[79]
S4 (1–16)	Denovo	ALWKTLLKKVLKAAAK	*Pseudomonas aeruginosa*	Disintegrates and release membrane lipids	[90]
P1	*Calliphora vicina*	FVDRNRIPRSNNGPKIPIISNP	*Escherichia coli, Staphylococcus aureus*, *Acinetobacter baumannii*	Degrades biofilm matrix	[88]
L-K6L9	Denovo	LKLLKKLLKKLLKLL	*Pseudomonas aeruginosa*	Degrades biofilms matrix	[52]
Piscidin-3	Fish	FIHHIFRGIVHAGRSIGRFLTG	*Pseudomonas aeruginosa*	Degrades eDNA	[91]
PI	Tick	PARKARAATAATAATAATAAT	*Streptococcus mutans*	Interferes and degrade EPS matrix	[87]
Hepcidin 20	Human	ICIFCCGCCHRSHCGMCCKT	*Staphylococcus epidermidis*	Acts on polysaccharide intercellular adhesin (PIA)	[88]
Nal-P-113	Denovo	AKR-Nal-Nal-GYKRKF-Nal-	*Porphyromonas gingivalis*	Down regulates genes related to transport and binding proteins	[99]
Human β-defensin 3 (hBD-3)	Human	GIINTLQKYYCRVRGGRCAVLSCLPKEEQIGKCSTRGRKCCRRKK	*Stahyloccocus epidermidis*	Targets icaA, icaD and icaR genes	[98]
DJK-5	Denovo	VQWRAIRVRVIR	*Pseudomonas aeruginosa*	Suppress spoT promoter activity	[96]
